# Polaritons in a Polycrystalline Layer of Non-fullerene Acceptor

**DOI:** 10.1021/jacs.2c11968

**Published:** 2023-01-23

**Authors:** Lixuan Liu, Zhixiang Wei, Stefan C. J. Meskers

**Affiliations:** † Molecular Materials and Nanosystems, Institute for Complex Molecular Systems, Eindhoven University of Technology, P.O. Box 513, Eindhoven, 5600 MB, The Netherlands; ‡ CAS Key Laboratory of Nanosystem and Hierarchical Fabrication, National Center for Nanoscience and Technology, Beijing100190, China; ¶ School of Future Technology, University of Chinese Academy of Sciences, Beijing100049, China

## Abstract

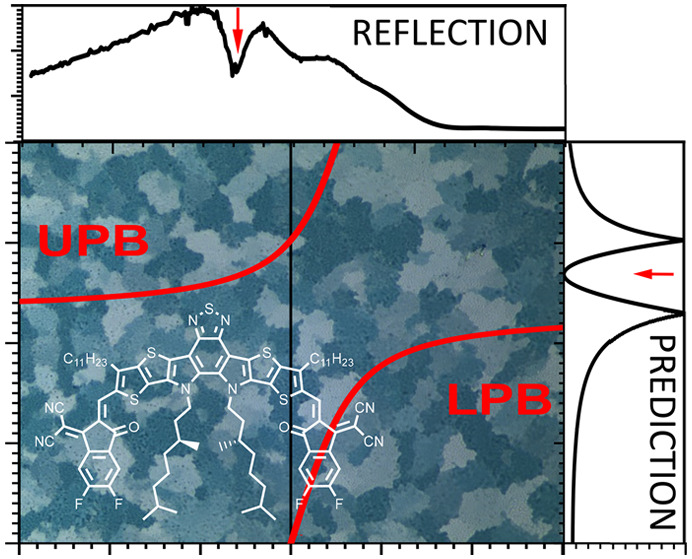

Non-fullerene acceptor molecules developed for organic solar cells feature a very intense absorption band in the near-infrared. In the solid phase, the strong interaction between light and the transition dipole moment for molecular excitation should induce formation of polaritons. The reflection spectra for polycrystalline films of a non-fullerene acceptor with a thienothienopyrrolo-thienothienoindole core of the so-called Y6 type indeed show a signature of polaritons. A local minimum in the middle of the reflection band is associated with the allowed molecular transition. The minimum in reflection allows efficient entry of light into the solid, resulting in a local maximum in external quantum efficiency of a photovoltaic cell made of the pure acceptor.

Polaritons are the quanta of light inside matter.^1,2^ A polariton is a quantum mechanical hybrid between a photon and an excitation in a material. The hybridization results from strong coupling between the excitation and resonant electromagnetic waves.^3^ Such strong coupling can be enforced by the confinement of light in an optical cavity incorporating also molecular material.^4−6^ Through the formation of such cavity polaritons, many amazing feats have been accomplished: low threshold lasing,^6−9^ optical transistors operating down to the single-quantum level,^10−13^ and external control over the rates of thermal chemical reactions inside the cavity.^14^ Strong coupling between light and matter can also be induced in the bulk condensed phase by tightly packing oriented dye molecules with large oscillator strength in, e.g., a molecular crystal or an aggregate.^13,15^ A spectroscopic signature of such bulk polaritons is the local minimum in the middle of the reflection band associated with an allowed electronic transition of the material.^16^

To support this view, we illustrate in Figure 1 the occurrence of this minimum for a variety of semiconductors. The explanation of the minimum is still under debate. It has been attributed to vibrations coupling with the excitation^17,18^ but has also been related directly to the dispersion relation of the polaritons.^16^ To see this, we first approximate the imaginary part of the refractive index or absorption by a delta function of frequency:
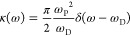
1with ω_D_ the resonance frequency of the allowed transition. ω_P_ denotes the plasma frequency, given by
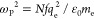
2where *N* is the number of molecules per unit volume, *f* the oscillator strength, *ε*_0_ the permittivity of vacuum, *q*_e_ the electron charge, and *m*_e_ the mass.^27^

**Figure 1:**
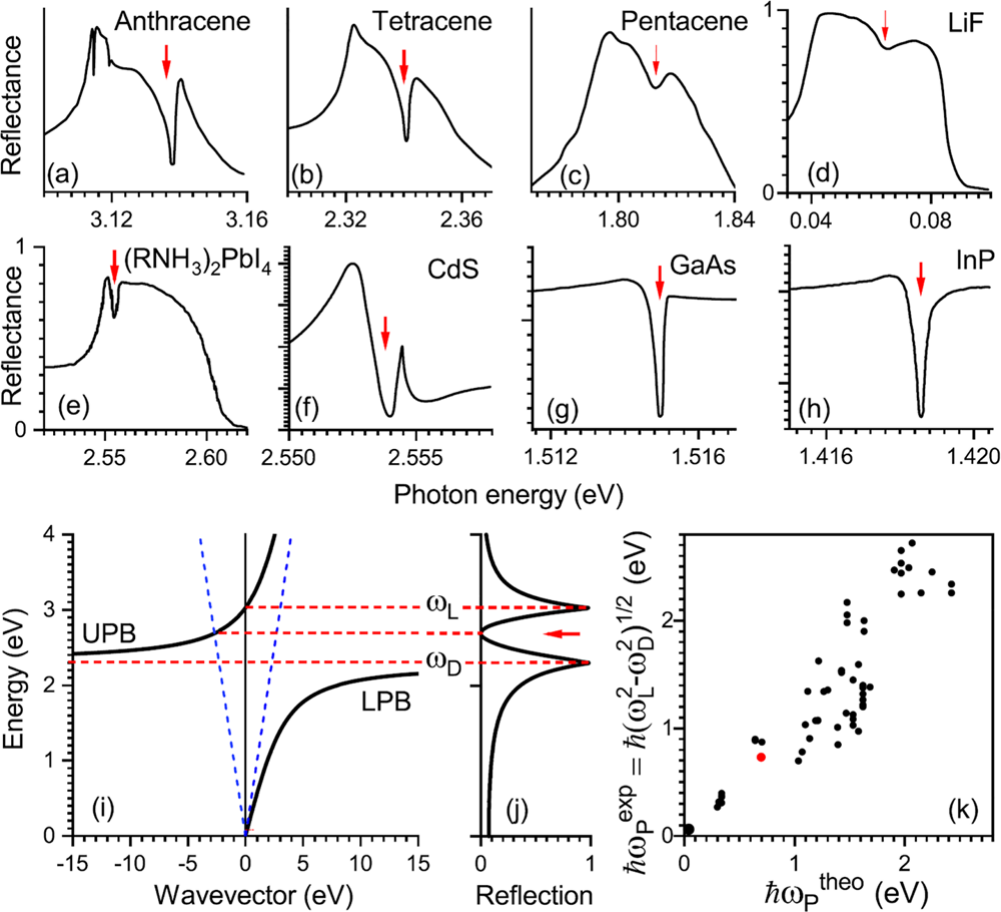
Experimental reflection spectra of organic and inorganic semiconductors as reported in the literature: (a) *T* = 2 K, (001), *E*||***b***;^19^ (b) 2 K, (001), *E*||***b***;^20^ (c) 1.85 K, (001), *E*||***b***;^21^ (d) 2 K, (001), *E*||***b***, *k*||***c***;^22^ (e) 4 K, *E*⊥**c**, *k*||***c***, R = H_21_C_10_;^23^ (f) 2 K, (100);^24^ (g) 2 K;^25^ and (h) 4 K.^26^ (i) Dispersion relation based on eq 2. (j) Reflection based on the dispersion relation. (k) Experimental versus predicted energy quanta associated with the plasma frequency. The red dot represents the result from this study.

Applying the Kramers–Kronig transform,^28,29^ we directly obtain for the frequency dependence of the refractive index:
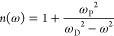
3Multiplying *n*(ω) with ω, we get the dispersion relation for the polariton with its characteristic upper and lower polariton branches (UPB and LPB); see Figure 1i. The reflection of light under normal incidence, *R*(ω) = ((1 – *n*(ω))/(1 + *n*(ω)))^2^, features two maxima, one at ω = ω_D_ and the other at ω_L_ = (ω_D_^2^ + ω_P_^2^)^1/2^, with a minimum in between; see Figure 1j. The minimum occurs at the frequency where the UPB crosses the photon line, i.e., where the electromagnetic waves in the material travel at the same phase velocity as in vacuum and therefore can enter the material unimpeded. The two maxima in reflection allow us to determine experimentally a value for the plasma frequency via ω_P_ = (ω_L_^2^ – ω_D_^2^)^1/2^. This value can then be compared to a theoretical prediction via eq 2. In Figure 1k we compare experimental and theoretical estimates for the energy quanta associated with the plasma frequency for a set of molecular crystals based on literature data (see Supporting Information (SI) for details).

The correlation between experimental and predicted values illustrates the usefulness of the polariton concept for understanding the optical properties of molecular crystals.^30,31^ Yet surprisingly little experimental evidence has appeared indicating a role for polaritons in organic optoelectronic devices.^32^ This may be related to the fact that often the molecular layers used are amorphous.^33−35^ Here we investigate a polycrystalline film of the non-fullerene acceptor (*S,S*)-BTP-4F (**1**).^36,37^ We show that, while for amorphous films hardly any polariton characteristics are observable, for thermally annealed polycrystalline films the reflection spectrum shows a pronounced minimum in the middle of the lowest band.

Solutions of **1** in chloroform feature an intense absorption centered around 732 nm, with decadic absorption coefficient ε = 0.979 × 10^5^ M^–1^ cm^–1^, and are characterized by an oscillator strength *f* = 0.7 and a transition dipole moment μ_01_ = 10.2 D; see Figure 2.

**Figure 2:**
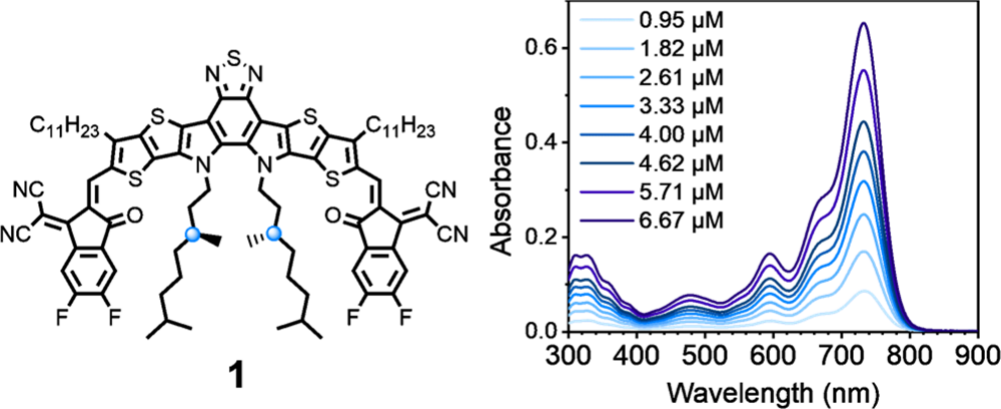
Structure of **1** and its absorption spectrum in chloroform at various concentrations with an optical pathlength of 1 cm.

Thermal annealing of a spin-coated layer of **1** results in a polycrystalline film; see Figure 3.^38,39^ Inspection of the domains of the film between parallel polarizers reveals a pronounced dichroism across the visible spectrum, indicating that all molecules are aligned in the same direction. Between crossed polarizers, the films show the expected birefringence associated with molecules aligned in the plane of the film. X-ray diffraction of a single crystal indicates packing of molecules in quasi-two-dimensional layers, separated by the long alkyl sidechains on the molecules.^40−45^ Within the quasi-2D layers, the dye molecules pack in a brickwork arrangement, with the transition dipole moments for the lowest allowed transition all pointing in the same direction.^46^

**Figure 3:**
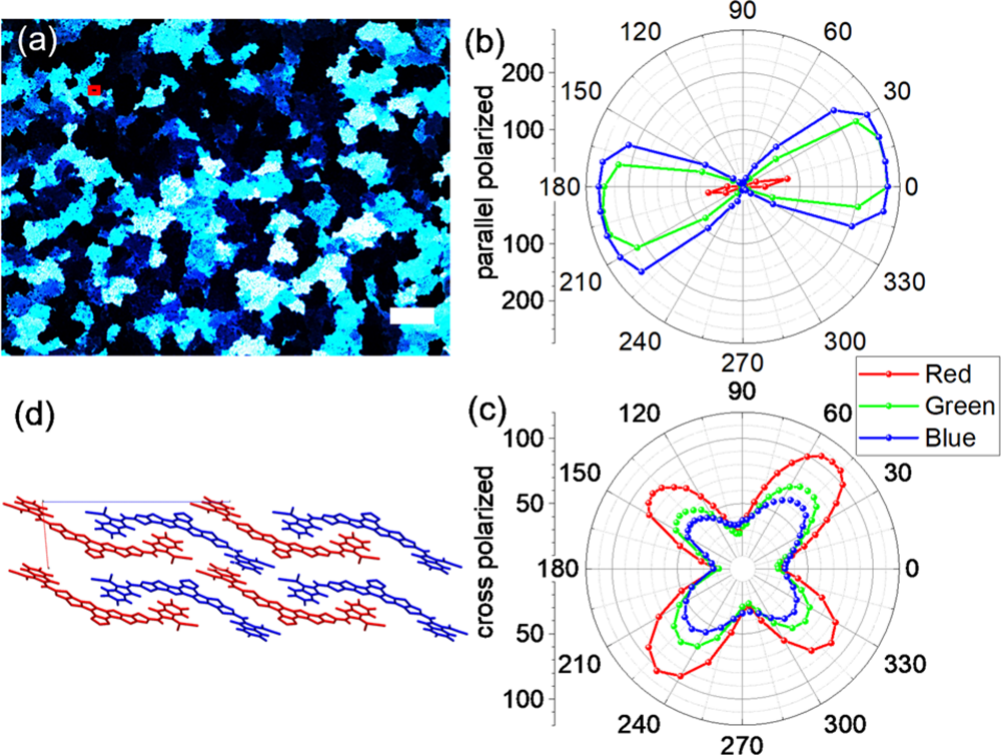
(a) Polarizing optical microscope image of a thermally annealed film of **1** between parallel polarizers. Scale bar: 40 μm. (b, c) Polar diagrams for (b) the transmitted red, green, and blue light for a single crystalline domain between parallel polarizes and (c) crossed polarizers. (d) Packing of molecules in quasi-2D layers in a crystal of **1**.

The polycrystalline film of **1** strongly reflects light with wavelength near 900 nm, with reflection close to 30% for near normal incidence; see Figure 4. Looking at the reflection graph starting at the long-wavelength side, we see an incease of the reflectivity with decreasing wavelength. This is to be expected because we approach the allowed lowest absorption band near 800 nm (see Figure 2), with the refractive index increasing sharply. Upon increasing the angle of incidence θ_i_, the reflectivity for *p*-polarized light at long wavelengths first decreases and then increases again for θ_i_ > 70°. This is consistent with the known vanishing reflectivity for *p*-polarized light when incident under the Brewster angle. The reflection spectrum shows a first maximum for light with wavelength λ = 952 nm and a second extremum at λ = 830 nm, with in between the characteristic minimum at λ = 875 nm. From the maxima we estimate ℏω_P_ = 0.73 eV. Using the oscillator strength *f* for **1** in solution and the number density in the crystal from X-ray diffraction, eq 2 predicts ℏω_P_ = 0.70 eV.

**Figure 4:**
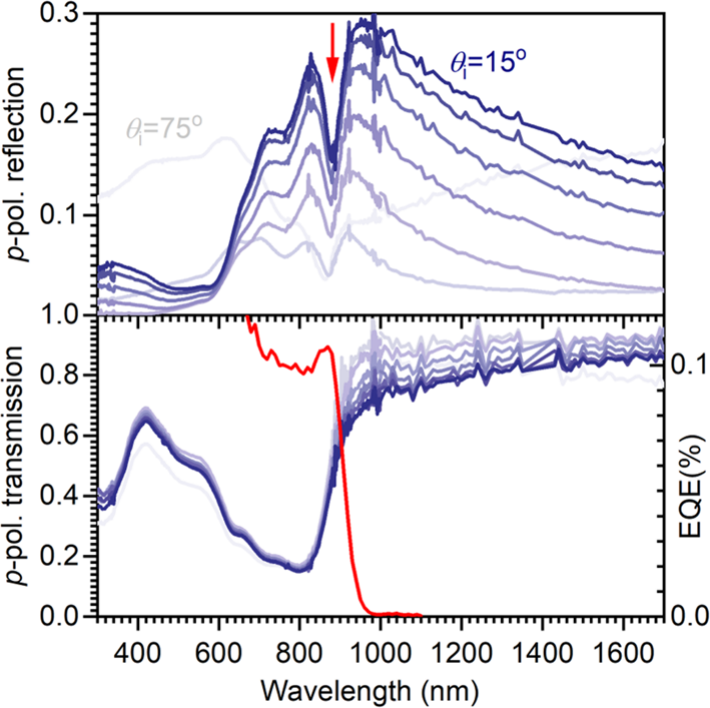
(Top) *p*-polarized reflection for a polycrystalline film of **1** annealed at 290 °C for various angles of incidence θ_i_ from 15° to 75°. (Bottom) *p*-polarized transmission (blue) for θ_i_ from 0° to 70° and external quantum efficiency (EQE, red) from a photovoltaic cell glass/ITO/ZnO/**1**/MoO_3_/Ag/sputtered ITO under 1 sun illumination, thermally annealed at 110 °C.

Films annealed at a lower temperature (110 vs 290 °C) show smaller domains. The reflection spectra for these films show maxima at the same characteristic wavelength but with a minimum that is less deep; see SI. Preliminary data on the relation between the depth of the reflection minimum and the typical lateral size of the crystalline domains indicate a lateral coherence length up to 35 μm for the polaritons generated in the film via the minimum in reflection (Figure S13).

Films annealed at 110 °C remain closed so that diodes without electrical shorts can be fabricated with a layer of **1** as semiconducting medium. We use semitransparent contacts to minimize any optical cavity effects. These diodes show a small but significant photovoltaic effect and generate photocurrent under simulated sunlight of about 1 sun intensity, with an external quantum efficiency (EQE) under short circuit exceeding 0.1% for light with wavelength around 800 nm; see Figure 4, bottom. The EQE spectrum shows a local maximum near 875 nm that coincides with the minimum in reflection for film annealed at 110 °C. From this we conclude that the local minimum in the reflection band allows light to enter the semiconducting molecular layer efficiently and to contribute to photocurrent generation.

In conclusion, polycrystalline films of **1** show the characteristic feature of polaritons in their reflection spectrum. The local minimum in the lowest reflection band allows efficient entry of light at the onset of the absorption band despite the refractive index in this region. This shows that polariton effects should be considered when evaluating optoelectronic properties of molecular semiconductors.

## Supporting Information Available

Experimental section, database of reflection spectra and crystal structure, crystallographic parameters of **1**, photovoltaic parameters, absorption coefficient in solution, optical microscope images, transmission and reflection spectra for films, EQE spectra, the dependence of relative depth of the reflection minimum on the lateral domain size, and GIWAXS patterns of thermally annealed films (PDF)
