# Supplementary material for: Polaritons in a Polycrystalline Layer of Non-fullerene Acceptor
Source: J Am Chem Soc. 2023 Jan 23;145(4):2040–4. doi: 10.1021/jacs.2c11968 (PMC9896558; doi:10.1021/jacs.2c11968)
Supplement: Supplementary file 1 — ja2c11968_si_001.pdf [file ja2c11968_si_001.pdf]

Supporting Information

Polaritons in a polycrystalline layer of non-fullerene acceptor.

Lixuan Liu[†‡¶], Zhixiang Wei[‡¶*], Stefan C.J. Meskers[†*].

†Molecular Materials and Nanosystems, Institute for Complex Molecular Systems, Eindhoven University of Technology, P. O. box 513, Eindhoven, NL 5600 MB, The Netherlands

‡CAS Key Laboratory of Nanosystem and Hierarchical Fabrication, National Center for Nanoscience and Technology, Beijing, 100190, China

¶School of Future Technology, University of Chinese Academy of Sciences (UCAS), Beijing, 100049, China

Table of Contents

Experimental Section 3

Supporting tables 4

Supporting figures 9

Supporting references 23

Experimental Section

1. Material Synthesis. The synthetic procedures for the enantiopure, chiral molecule **1**, also known as (*S,S*)-BTP-4F, can be found in our previous reports.[1-2] The structure and molecular weight were confirmed via nuclear magnetic resonance (NMR) (AVANCE III HD 400) and matrix-assisted laser desorption ionization–time-of-flight mass spectrometry.

2. Photovoltaic cell fabrication. Glass substrates with patterned ITO (Naranjo Substrates) were cleaned by sonication in acetone, soap water, deionized water, 2-propanol in order. Then the substrates were dried and treated in an ultraviolet (UV)–ozone oven for 30 min.

Photovoltaic cells were made with an architecture of glass/ITO/ zinc oxide(ZnO)/(*S,S*)-BTP-4F/MoO_3/Ag/sputtered ITO. Firstly, ZnO nanoparticle precursor was spin coated at 4000 rpm onto the ITO substrate and annealed at 200 °C for 30 min. Then, pure (*S,S*)-BTP-4F with a concentration of 24 mg mL^{-1} was spin coated at a rotatory speed of 1000rpm to form the active layer. Finally, 15 nm MoO_x and 15 nm Ag layer were thermally evaporated under a high vacuum (*ca*. 1×10^{-6} mbar), followed by 180 nm sputtered ITO back electrodes made via radio frequency magnetron sputtering.

3. Current density-Voltage (*J–V*) measurements. *J–V* characteristics were measured using a Keithley 2400 source meter. A tungsten–halogen lamp combined with a Schott GG385 UV filter and a Hoya LB120 daylight filter was used to stimulate the AM 1.5 G (100 Mw cm^{-2}) illumination. Light intensity was calibrated by a Si photodiode.

4. External quantum efficiency (EQE) characteristics. The light from a 50 W tungsten–halogen lamp (Philips focusline) was chopped at 158 Hz before passing into a monochromator (Oriel, Cornerstone 130) to illuminate the cell. Si photodiode was used as reference to calibrate the current, which was fed into a current preamplifier (Stanford Research, SR 570). The voltage was measured using a lock-in amplifier (Stanford Research, SR830). A green light emitting diode (Thorlabs, M530L3) was used as a light bias to generate approximately 1 Sun illumination intensity.

5. Optical properties. Absorption spectra were measured using a PerkinElmer Lambda 1050 UV-vis-NIR spectrophotometer. Ellipsometry spectra were measured using a WVASE31 ellipsometer (J.A. Woollam Co.).

Supporting tables

1. Database of reflection spectra and crystal structure.

Table S1 Chemical compound (C, See Scheme S1), oscillator strength f , code for crystal structure in the Cambridge Structural Database, number of molecules in the unit cell Z, reflecting plane, ratio of the dipole strength for the electronic transition from the ground state to the various Davydov levels, resonance frequency for isolated molecules ω_D , plasma frequency calculated from oscillator strength and crystal structure ω_P^{calc}, experimentally determined value from the two reflection maxima at ω_D and ω_L for the plasma frequency: $(\omega_L^2-\omega_D^2)^{1/2}$, angle between the main transition dipole moment and the normal of the reflecting surface χ, ratio of the reflectance at the lower frequency maximum ω_D and the high frequency maximum ω_L, R_D/R_L

**C**	f	Crystal	Z	plane	μ_a^2/μ_b^2 (a)	ω_D	ω_P^{calc}	$(\omega_L^2-\omega_D^2)^{1/2}$	χ	R_D/R_L
						eV	eV	eV	deg.	
**1**	1.10	ROQMAL[3]	2	(010)	2:0	2.33	1.97	2.53	88	1.07 [4]
				(110)		2.27	1.97	2.65	70	1.23 [4]
				(100)		2.44	1.97	2.44	60	0.74 [4]
				(011)		2.50	1.97	2.24	35	0.44 [4]
**2**	1.10	YOHRES[5]	2	(010)	2:0	2.32	1.43	1.53	82	0.92 [6]
				(010)	(15K)	2.40	1.43	1.52	82	0.91 [6]
				(001)		2.40	1.43	1.54	52	0.39 [6]
				(100)		2.40	1.43	1.54	42	0.26 [6]
**3**	1.10	FIXJEBO1[7]	2	(001)	2:0	1.99	3.17	2.47	89	1.14 [8]
				(110)		2.00	3.27	2.59	66	0.58 [8]
**4**	1.08	CPIMPM[9]	2	(110)	2:0	1.77	1.48	2.05	81	1.11 [10]
				(100)		1.77	1.48	2.17	34	0.12 [10]
				(010)		1.88	1.48	1.98	63	0.56 [10]
				(10$\bar{1}$)		1.78	1.48	2.05	49	0.58 [10]
**5**	1.06	DEPICY[11]	4	(100)	3.3:0.7:0:0	2.13	1.58	1.59	40	0.74 [12]
				(1$\bar{2}$0)		2.26	1.58	0.97	51	0.77 [12]
				(100)		2.26	0.70	0.87	89	4.52 [12]
	1.06	PSISO priv[13]	4	(01$\bar{1}$)	2.9:0.71:0.25:0.16	2.19	1.47	1.14	81	1.16 [13]
**6**	1.06	DAQYEZ[14,13]	4	(100)	3.2:0.6:0.17:0.05	2.23	1.53	1.09	85	1.20 [13]
				($\bar{1}$01)		2.29		1.03	41	0.76 [13]
				(101)		2.29		1.45	54	0.62 [13]
				(110)		2.22		1.13	89	1.19 [13]
**7**	1.0	DECYNI[15]	4	(100)	3.8:0.2:0:0	2.28	1.62	1.38	35	0.65 [16]
				($\bar{1}$02)		2.28		1.32	79	1.42 [16]
				(100)		2.28		1.40	35	0.60 [12]
				(011)		2.15		1.22	62	0.83 [12]
				(01$\bar{1}$)		2.16		1.27	60	0.80 [12]
				(100)		2.38		1.20	35	0.57 [17]

**8**	0.023	ANTCEN09[18]	2	(001)	1.6:0.4	3.12	0.34	0.40	90	1.13[19]
				(ī ī2)		3.12	0.34	0.31	47	0.84 [20]
				(ī ī 1)		3.12	0.34	0.36	36	0.58 [20]
				(ī ī 0)		3.12	0.34	0.37	41	0.32 [20]
	0.98[21]			(010)	2:0	4.96	2.42	2.34	90	1.01 [22]
				(001)	2:0	4.96	2.42	2.26	30	0.06 [22]
**9**	0.025	TETCEN01[23]	2	(001)	1.6:0.4	2.32	0.31	0.32	75	1.51 [24,25]
**10**	0.028	PENCEN[26]	2	(001)		1.80	0.30	0.27	75	1.23 [27]
**11**	1.22	PEWXAQ01[28]	4	(100)	4:0:0:0	2.70	2.15	2.26	25	0.89 [29]
**12**	1.65	PORZIO[30]	4	(100)		2.47	2.25	2.45	20	0.64 [31]
**13**	2.10	ZAQZUM[32]	4	(100)		2.30	2.04	2.49	24	0.50 [33]
**14**	0.67	BIPHEN04[34]	2	(001)	2:0	5.1	2.07	2.72	17	0.41 [35]
**15**	0.33	PERLEN (β)[36]	2	(110)	1.99:0	2.63	1.19	1.07	79	1.35 [37]
		PERLEN01 (α)[38]	2	(110)		2.65	1.21	1.08	78	1.08 [39]
			2	(ī 10)		2.63	1.21	1.07	78	1.35 [37]
**16**	1	UGOMIM[40]	4	(010)	3.7:0.3:0:0	2.49	1.39	0.85	90	0.99 [41]
		YAMMAB[42]	2	(010)	1.3:0.3	2.21	1.10	1.03	90	0.93 [41]
		YAMMUV[43]	1	(001)	1	2.03	1.22	1.62	73	1.34 [41]
**17**	1	AFOMOX[44]	2	(012)	1.98:0.02	2.2	1.39	1.01	68	1.09 [45]
**18**	1	DICPAG[46]	2	(102)	1.8:0.2	2.12	1.69	1.38	90	1.21 [45]
**19**	0.5	VAGPEZ[47]	2	(001)	2:0	2.09	1.30	1.35	89	1.87 [47]
**20**	0.5	XODFAX[48]	2	(0 ī 0)	2:0	2.19	1.14	0.91	88	1.23 [49]
**21**	0.5	OHIXAE[47]	2	(001)	2:0	2.11	1.27	1.34	88	1.23 [47]
**22**	1	PIJHIA01[50]	4	(010)	3.0:0.5:0.3:0.2	2.22	1.13	1.34	27	0.42[51]
**23**	0.11	SATTIR[52]	1	(001)	-	2.23	0.64	0.89	86	2.09 [53]
**24**	0.11	MAMGUD01[54]	1	(001)	-	2.11	0.64	0.90	82	5.59 [55]
**25**	0.55	FONFAQ01[56]	1	(100)	-	1.77	1.07	0.78	63	1.75 [57]
	0.55	FONFAQ[58]	4	(010)	3.87:0.07:0.00.04:0.02	1.80	1.04	0.70	90	1.90 [57]
**26**	1.01	VAYSET01[59]	1	(100)	-	1.66	1.63	2.00	63	0.90 [60]
			1	(1 ī 0)	-	1.63	1.63	1.90	89	0.95 [60]
**27**	1	LiF	1	(100)		0.050	0.041	0.056	0	0.90 [61]

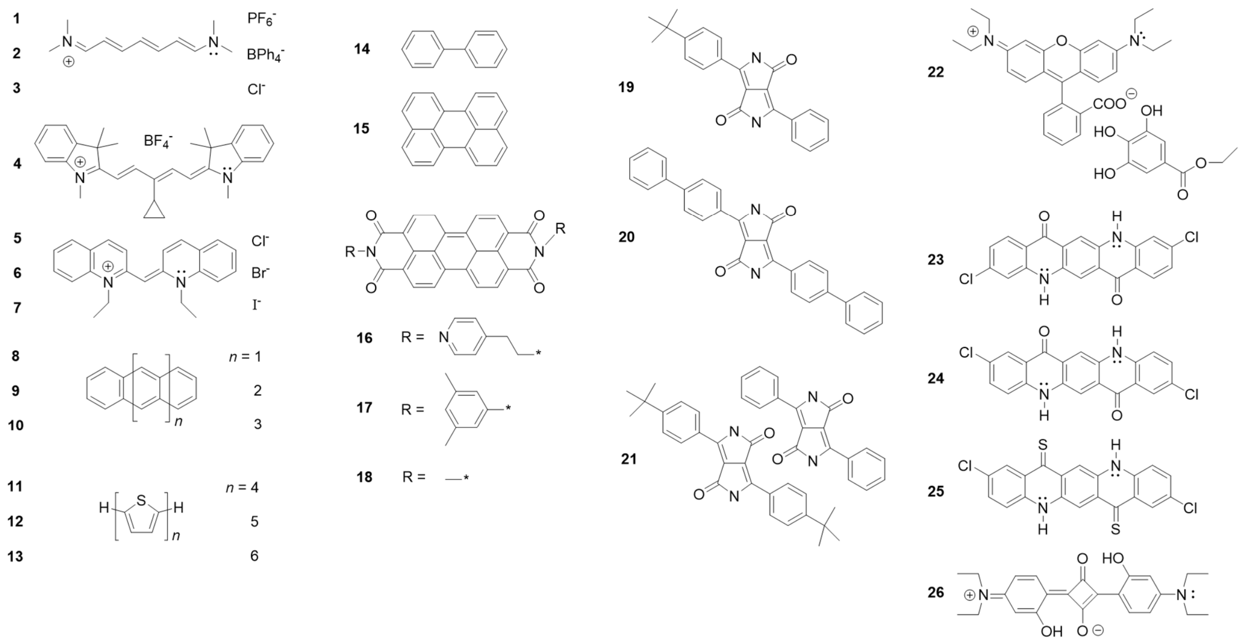

Scheme S1. Molecular structers in Table S1.

Table S2. Crystallographic parameters of **1** ((*S,S*)-BTP-4F)

Molecule		**1** ((*S,S*)-BTP-4F)
Formula		C_{86} H_{94} F_4 N_8 O_2 S_5
Space group		P $\overline{1}$ (2)
	a	8.2930(5)
cell length	*b*	18.6556(11)
	c	25.9225(15)
	α	87.763(3)
cell angel	β	84.137(3)
	γ	82.117(3)
cell volume		3950.54
Z, Z'		Z: 2 Z': 0

Table S3. Photovoltaic parameters of single-component photovoltaic cells

**1** ((*S*,*S*)-BTP-4F)	Rotatory speed (rpm)	TA	V_{OC} (V)	J_{SC} (mA cm^{-2})	FF (%)	PCE (%)
24 mg/ml	S1 1000	no	0.57	0.0161	33	0.0030
	S3 1000	110 °C	0.76	0.0240	37	0.0067
20 mg/ml	S4 1000	110 °C	0.75	0.0313	38	0.0089

Supporting figures

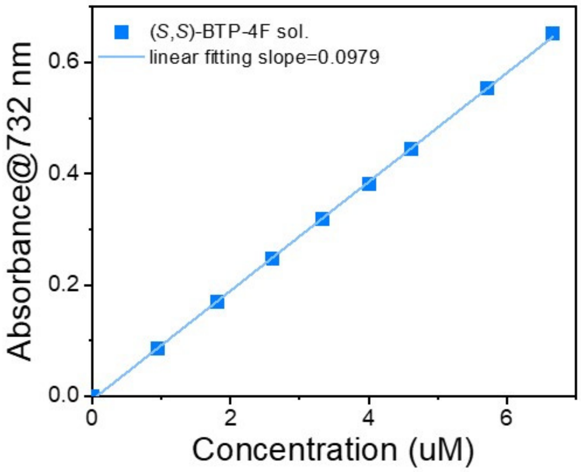

Figure S1 The absorbance of **1** ((*S*,*S*)-BTP-4F) in chloroform solution at 732 nm with various concentration. The slope of the linear fit to the datapoints yields the absorption coefficient.

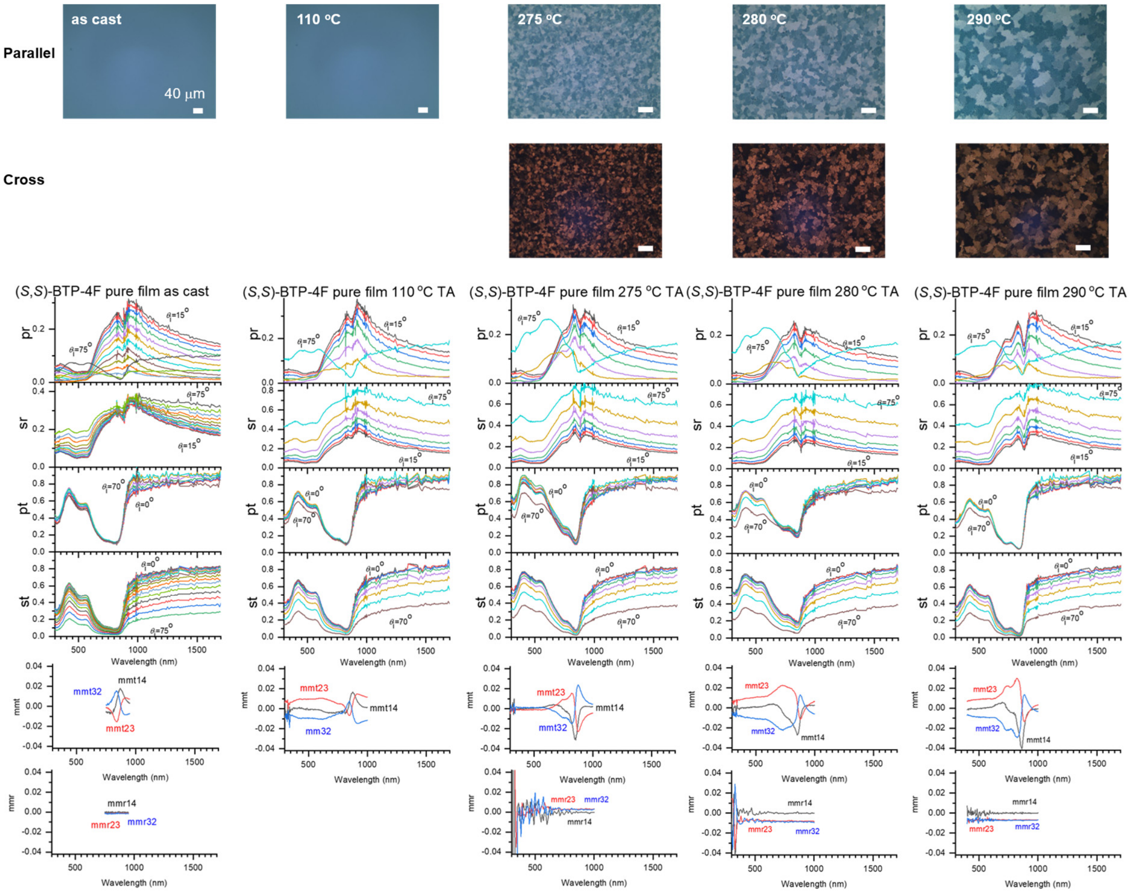

Figure S2 Optical polarization microscope images, reflection, transmission and selected mueller matrix element spectra for films of **1** ((*S*,*S*)-BTP-4F) with thickness *ca.* 100 nm. From left to right : as cast film and films thermally annealed at 110 °C, 275 °C, 280 °C, and 290 °C. pr (sr): *p(s)*-polarized reflection. pt (st): *p*-(*s*-) polarized transmission. The lower two panels in each row show selected mueller matrix elements in transmission (mmt) and reflection (mmr). No difference in reflection of left and right polarized light is observed ; the mm23 and mm32 elements suffer from a constant, instrument related offset from zero.

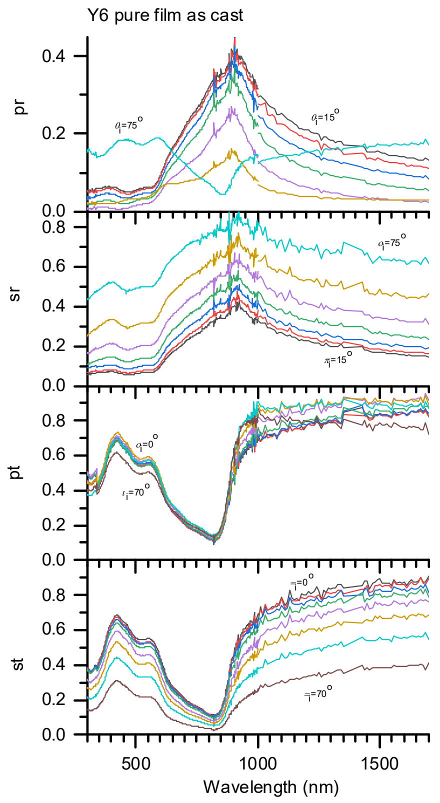

Figure S3 Transmission and reflection spectra of as cast film with thickness *ca.* 100 nm of Y6, i.e. the derivative of **1** with racemic 2-ethyl-hexyl side chains instead of the two *S*-citronellol derived N-substituents.

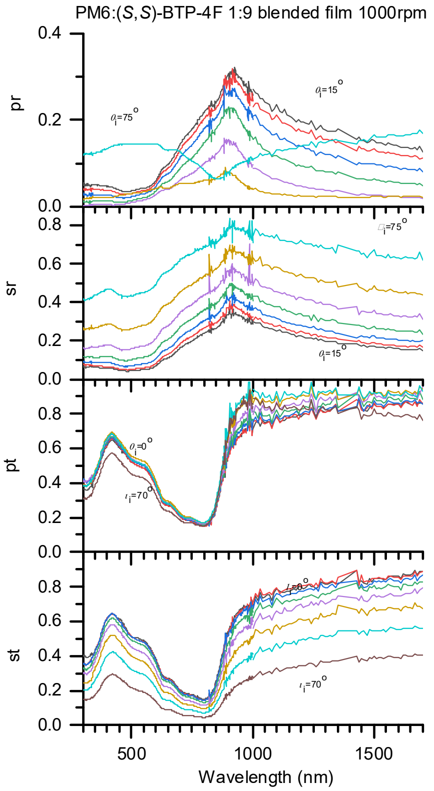

Figure S4 Transmission and reflection spectra of blended films of PM6 : **1** ((*S*,*S*)-BTP-4F) with 1:9 weight ratio[1,2] and with a thickness of *ca*. 120 nm.

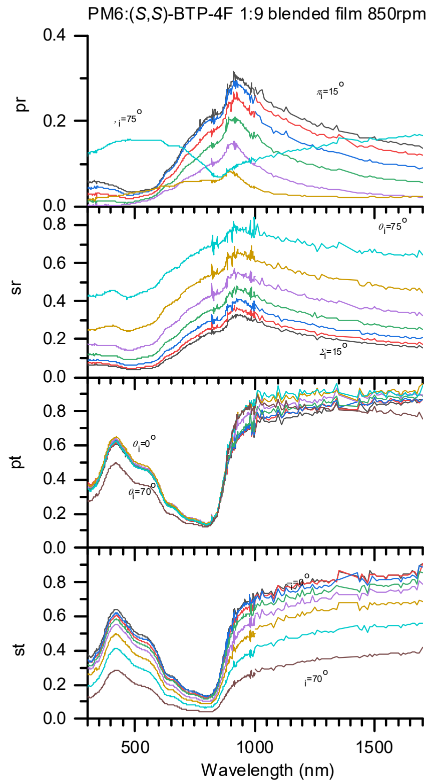

Figure S5 Transmission and reflection spectra of PM6 : **1** ((*S*,*S*)-BTP-4F) blended films with 1:9 weight ratio and thickness of *ca*. 140 nm.

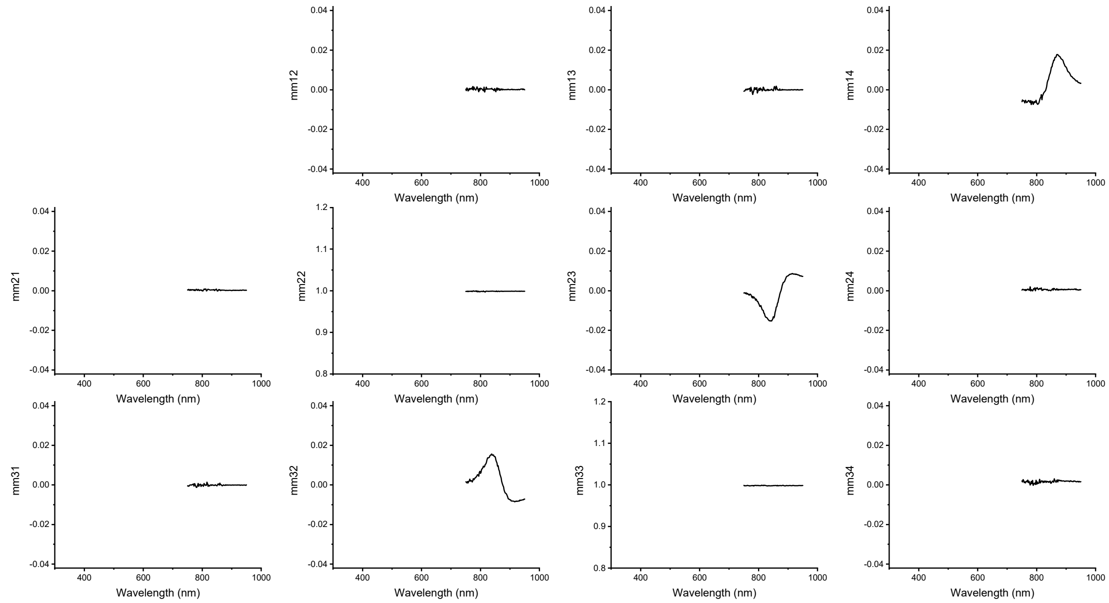

Figure S6 As measured Mueller matrix data for transmission measurements on an as cast film of **1** ((*S*,*S*)-BTP-4F) .

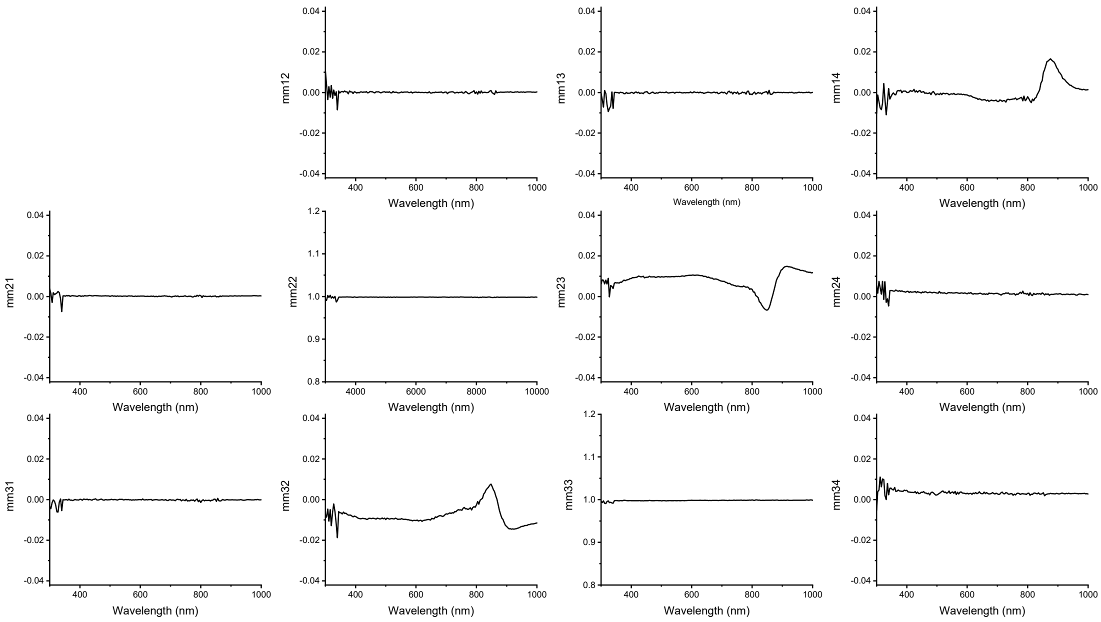

Figure S7 As measured Mueller matrix data for transmission measurements on a film of **1** ((*S*,*S*)-BTP-4F) thermally annealed at 110 °C.

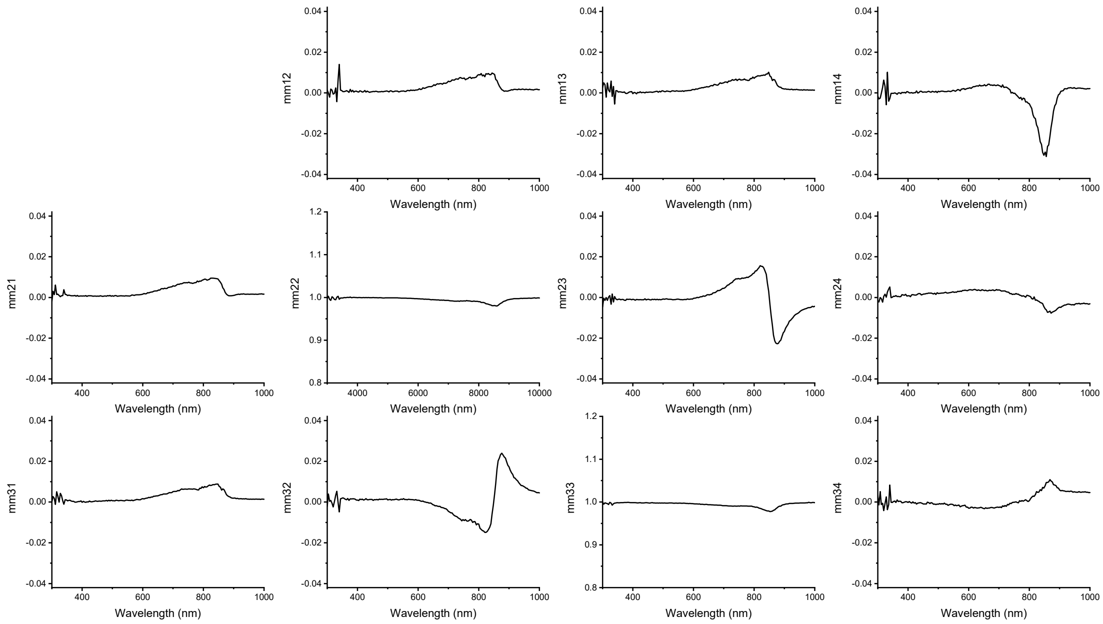

Figure S8 As measured Mueller matrix data for transmission measurements on a film of **1** ((*S*,*S*)-BTP-4F) thermally annealed at 275 ^{o}C .

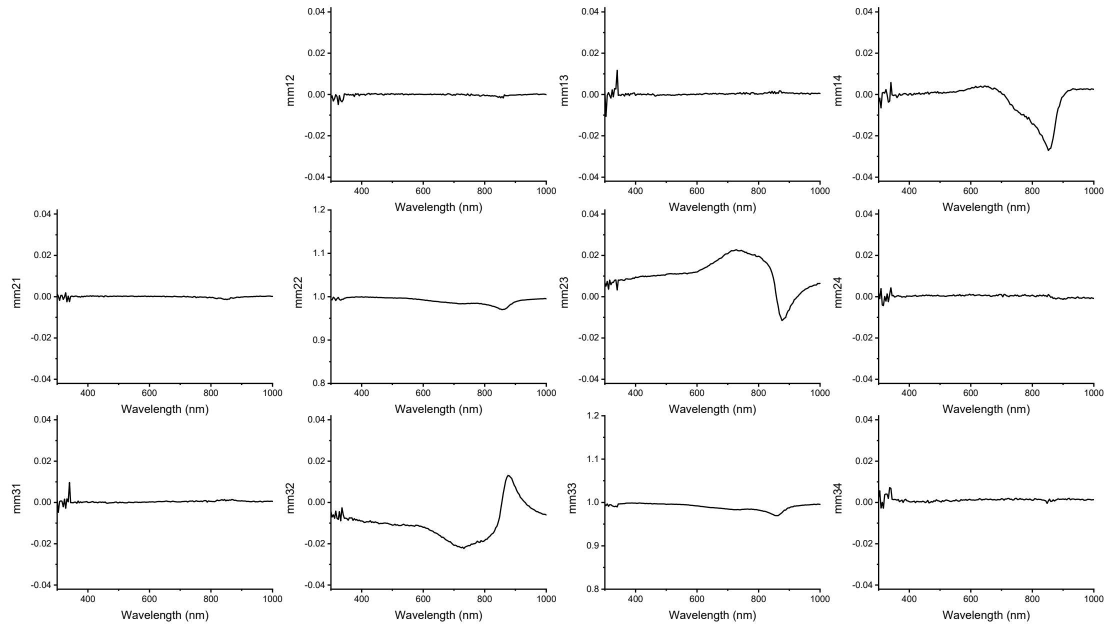

Figure S9 As measured Mueller matrix data for transmission measurements on a film of **1** ((*S*,*S*)-BTP-4F) thermally annealed at 280 ^{o}C .

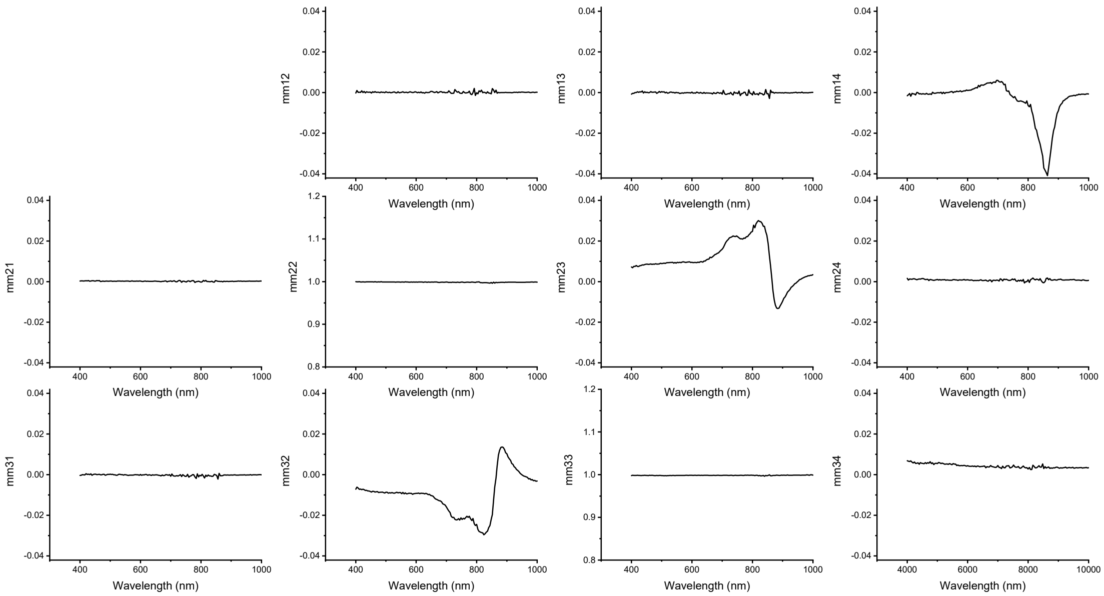

Figure S10 As measured Mueller matrix data for transmission measurements on a film of **1** ((*S*,*S*)-BTP-4F) thermally annealed at 290 °C.

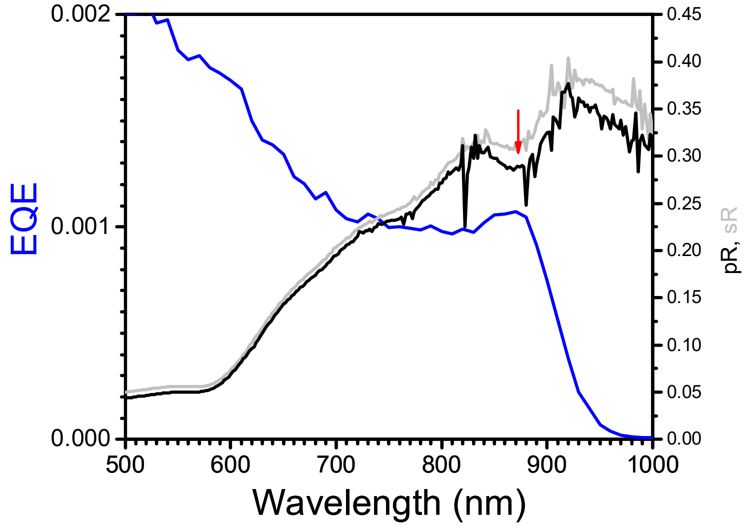

Figure S11 *p*- and *s*-polarized reflection for film of **1** annealed at 110 °C for an incidence θ_i of 15°, and External Quantum Efficiency (EQE) from a photovoltaic cell glass/ITO/ZnO/1/MoO_3/Ag/sputtered ITO under 1 sun illumination.

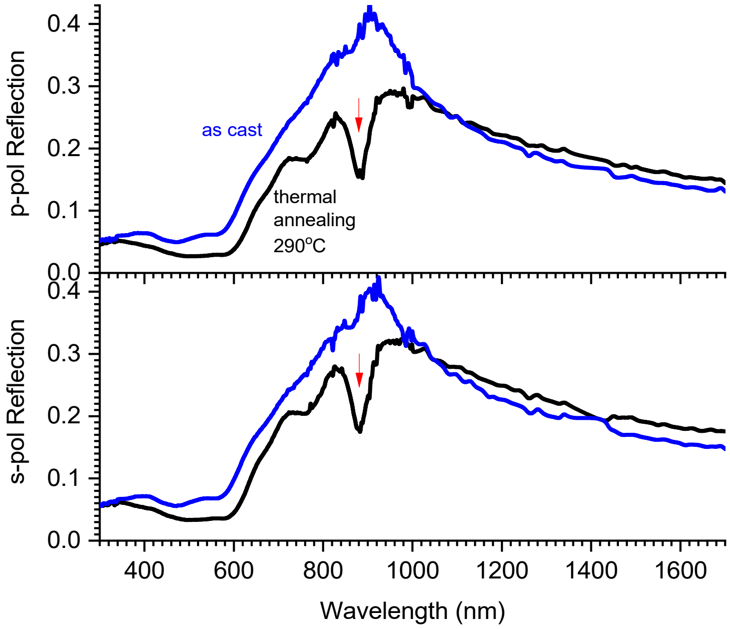

Figure S12. Comparison between reflection of a cast film of **1** (blue) and a film of **1** thermally annealed at 290°C (black). The top panel shows *p*-polarized reflection for an angle of incidence θ_i = 15°. The lower panel shows the corresponding *s*-polarized reflection.

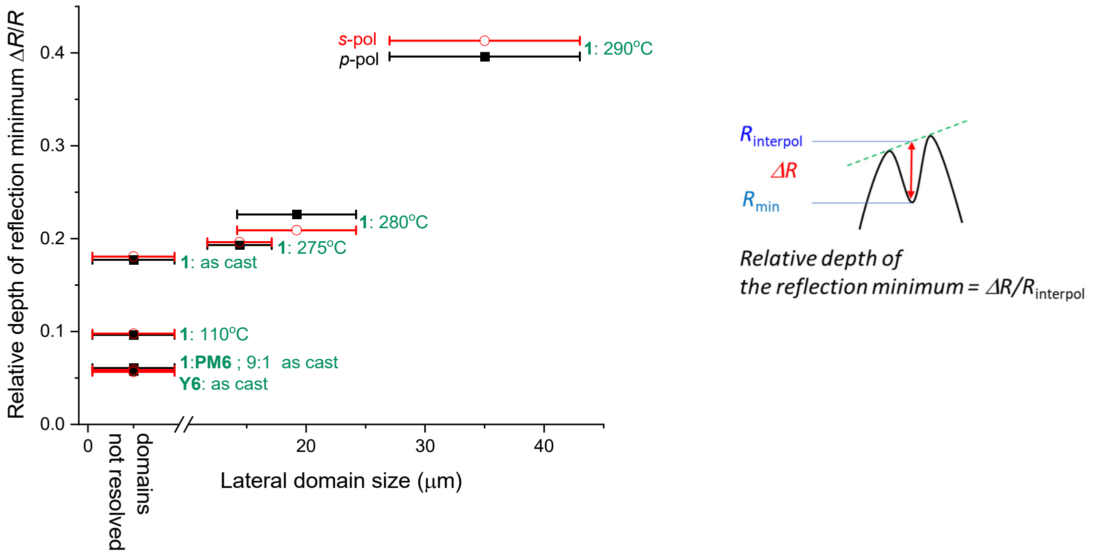

Figure S13. Relative depth of the minimum in *s*- and *p*-polarized reflection at q_i = 15° angle of incidence plotted as function of the typical lateral crystalline domain size for various films of non-fullerene acceptor. The lateral domain size was estimated from optical polarization microscope images with parallel polarizers as shown in Figure S2. Shown are data for films of pure 1 annealed at different temperatures. The horizontal error bars indicate the standard deviation in the domain size (*N*=38). For films of **1** as cast and annealed at 110°C, no domains are visible through the microscope and hence the domain size can only be estimated < 10 mm. Also films of 1 mixed with 10 wt % of PM6 polymer[1] and films of Y6, i.e. the derivative of **1** with racemic 2-ethyl-hexyl side chains instead of the two *S*-citronellol derived N-substituents, have been measured.

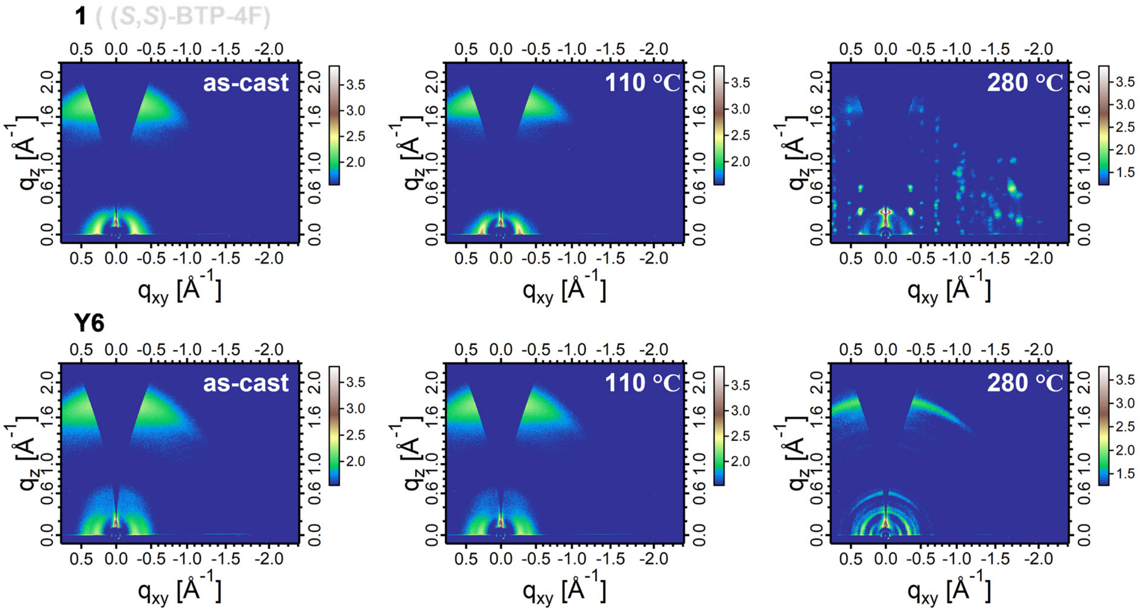

Figure S14. Grazing incidence wide angle X-ray scattering (GIWAXS) of films of **1** annealed at different temperatures (top row) and of Y6 (i.e. the derivative of 1 with racemic 2-ethyl-hexyl side chains instead of the two *S*-chiral citronellol derived N-substituents, bottom row).

Supporting references

(1) Liu, L.; Wei, Z.; Meskers, S. C. J., Semi-Transparent, Chiral Organic Photodiodes with Incident Direction-Dependent Selectivity for Circularly Polarized Light. *Adv. Mater.* doi.org/10.1002/adma.202209730.

(2) Liu, L.; Yang, Y.; Zhu, L.; Zhang, J.; Chen, K.; Wei, Z., Chiral Non‐Fullerene Acceptor Enriched Bulk Heterojunctions Enable High‐Performance Near‐Infrared Circularly Polarized Light Detection. *Small* **2022,** *18* (31), 2202941.

(3) Dahne, L.; Zobel, D.; Reck, G., One-dimensional staircase aggregates in crystals of 1,7-bis(dimethylamino)heptamethinium hexafluorophosphate, a polymethine dye. *Z. Krystallog.* **1997,** *212* (7), 529-531.

(4) Dahne, L., Polaritons in highly ordered cyanine dye films. *Mater. Sci. Eng. C* **1999,** *8-9*, 395-399.

(5) Dahne, L.; Reck, G., Deformation of polymethine structures by intermolecular interactions. *Angew. Chem. Int. Ed.* **1995,** *34* (6), 690-692.

(6) Dahne, L.; Horvath, A.; Weiser, G., influence of aggregation on the optical-spectra of a polymethine dye in single-crystals. *Chem. Phys.* **1995,** *196* (1-2), 307-316.

(7) Groth, P., On the disordered crystal-structure of bis(dimethylamino)-heptamethinium chloride tetrahydrate at room-temperature. *Acta Chem. Scand. B* **1987,** *41* (7), 547-550.

(8) Dahne, L.; Horvath, A.; Weiser, G.; Reck, G., The Chameleon dye 1,7-bis(dimethylamino)heptamethine. *Adv. Mater.* **1996,** *8* (6), 486-490.

(9) Allmann, R.; Debaerdemaeker, T.; Ferwanah, A. R.; Pressler, W.; Reichardt, C., Syntheses with substituted malonaldehydes .23. "gamma-cycloalkylpentamethinecyanine dyes. *Chem. Ber.* **1976,** *109* (9), 3005-3016.

(10) Hesse, H. J.; Fuhs, W.; Weiser, G.; Szentpaly, L. V., Directional dispersion of an exciton polariton in a pentamethinium cyanine dye. *Phys. Stat. Solidi B* **1976,** *76* (2), 817-826.

(11) Dammeier, B.; Hoppe, W., Crystal and molecular structure of n,n'-diethylpseudoisocyanin chloride. *Acta Cryst. B* **1971,** *27* (DEC15), 2364-2370.

(12) Tanaka, J.; Tanaka, M.; Hayakawa, M., Electronic-spectra of single-crystals of 1,1'-diethyl-2,2'-cyanine iodide, bromide, and chloride. *Bull. Chem. Soc. Jpn.* **1980,** *53* (11), 3109-3119.

(13) von Berlepsch, H.; Moller, S.; Dahne, L., Optical properties of crystalline pseudoisocyanine (PIC). *J. Phys. Chem. B* **2001,** *105* (24), 5689-5699.

(14) Koch, O.; Lork, E.; Kleemeier, M.; Wiegand, S.; Schröer, W., Crystal structure of 1-ethyl-2-(1-ethyl-H-quinoline-2-ylidenmethylene)- quinolinium nitrate monohydrate, $(C_{23}H_{25}N_2)NO_3 \cdot H_2O$. *Z. Kristallogr.-New Cryst. Struct.* **1999,** *214* (4), 567-568.

(15) Nakatsu, K.; Yoshioka, H.; Morishita, H., Structure of 1,1'-diethyl-2,2'-cyanine iodide, a photographic sensitizing dye. *Acta Crystallogr. B* **1977,** *33* (JUL15), 2181-2188.

(16) Marchetti, A. P.; Salzberg, C. D.; Walker, E. I. P., Optical-properties of crystalline 1,1'-diethyl-2,2'-cyanine iodide. *J. Chem. Phys.* **1976,** *64* (11), 4693-4698.
(17) Delaney, J.; Morrow, M.; Eckhardt, C. J., Observation of the j-band in the crystal spectra of pseudoisocyanine (pic). *Chem. Phys. Lett.* **1985,** *122* (4), 347-351.
(18) Brock, C. P.; Dunitz, J. D., Temperature-dependence of thermal motion in crystalline anthracene. *Acta Crystallogr. B* **1990,** *46*, 795-806.
(19) Brodin, M.; MA, D.; Marisova, S., Special features of surface-exciton and volume-exciton spectra of an anthracene crystal in case of different wave-vector directions. *Opt. Spectrosc.* **1973,** *34* (6), 1120-1125.
(20) Syassen, K.; Philpott, M. R., Reflection spectra of natural faces of crystalline anthracene. *J. Chem. Phys.* **1978,** *68* (11), 4870-4874.
(21) Ferguson, J.; Reeves, L. W.; Schneider, W. G., Vapor absorption spectra and oscillator strengths of naphthalene, anthracene, and pyrene. *Can. J. Chem.* **1957,** *35* (10), 1117-1123.
(22) Koch, E. E.; Otto, A., Optical-properties of anthracene single-crystals in excitonic region of spectrum between 4 and 10.5 ev. *Chem. Phys.* **1974,** *3* (3), 370-383.
(23) Holmes, D.; Kumaraswamy, S.; Matzger, A. J.; Vollhardt, K. P. C., On the nature of nonplanarity in the N phenylenes. *Chem.-Eur. J.* **1999,** *5* (11), 3399-3412.
(24) Turlet, J. M.; Philpott, M. R., Surface and bulk exciton-transitions in reflection spectrum of tetracene crystals. *J. Chem. Phys.* **1975,** *62* (11), 4260-4265.
(25) Kolendritskii, D. D.; Kurik, M. V.; Piryatinskii, Y. P., Exciton reflection spectra of tetracene single-crystals. *Phys. Stat. Sol. B* **1979,** *91* (2), 741-751.
(26) Campbell, R. B.; Trotter, J.; Monteath.J, Crystal structure of hexacene, and a revision of crystallographic data for tetracene and pentacene. *Acta Crystallogr. B* **1962,** *15* (3), 289-290.
(27) Piryatinski, Y. P.; Kurik, M. V., Influence of structural defects on excitonic photoluminescence of pentacene. *Ukr. J. Phys.* **2011,** *56* (10), 1048-1055.
(28) Siegrist, T.; Kloc, C.; Laudise, R. A.; Katz, H. E.; Haddon, R. C., Crystal Growth, Structure, and Electronic Band Structure of α-4T Polymorphs. *Adv. Mater.* **1998,** *10* (5), 379-382.
(29) Tavazzi, S.; Campione, M.; Laicini, M.; Raimondo, L.; Borghesi, A.; Spearman, P., Measured Davydov splitting in oligothiophene crystals. *J. Chem. Phys.* **2006,** *124* (19), 194710.
(30) Porzio, W.; Destri, S.; Mascherpa, M.; Bruckner, S., Structural aspects of oligothienyl series from x-ray-powder diffraction data. *Acta Polym.* **1993,** *44* (6), 266-272.
(31) Tavazzi, S.; Laicini, M.; Sassella, A.; Spearman, P., Reflectance spectra of quinquethiophene single crystals. *Synth. Metals* **2003,** *139* (3), 873-875.
(32) Horowitz, G.; Bachet, B.; Yassar, A.; Lang, P.; Demanze, F.; Fave, J. L.; Garnier, F., Growth and characterization of sexithiophene single-crystals. *Chem. Mater.* **1995,** *7* (7), 1337-1341.

(33) Weiser, G.; Moller, S., Directional dispersion of the optical resonance of pi-pi* transitions of alpha-sexithiophene single crystals. *Phys. Rev. B* **2002,** *65* (4), 045203.
(34) Charbonneau, G. P.; Delugeard, Y., Structural transition in polyphenyls .5. biphenyl - 3-dimensional data and new refinement at 293 k. *Acta Cryst. B* **1977,** *33* (MAY13), 1586-1588.
(35) McLaughlin, T. G.; Clark, L. B., Electronic-spectrum of biphenyl. *Chem. Phys.* **1978,** *31* (1), 11-18.
(36) Tanaka, J., The electronic spectra of aromatic molecular crystals .2. the crystal structure and spectra of perylene. *Bull. Chem. Soc. Jpn.* **1963,** *36* (10), 1237-1249.
(37) Tanaka, J.; Kishi, T.; Tanaka, M., Electronic-spectra of perylene crystals. *Bull. Chem. Soc. Jpn.* **1974,** *47* (10), 2376-2381.
(38) Camerman, A.; Trotter, J., Crystal and molecular structure of perylene. *Proc. R. Soc. London. Ser. A* **1964,** *279* (1376), 129-146.
(39) Fuke, K.; Kaya, K.; Kajiwara, T.; Nagakura, S., Polarized reflection and absorption-spectra of perylene crystals in monomeric and dimeric forms. *J. Mol. Spectrosc.* **1976,** *63* (1), 98-107.
(40) Mizuguchi, J.; Tojo, K., Crystal structure ofN,N¢-bis(2-(4-pyridyl)ethyl)perylene-3,4:9,10bis(dicarboximide), $C_{38}H_{24}N_4O_4$. *Z. Kristallogr.* **2002,** *217*, 247–248.
(41) Mizuguchi, J.; Hino, K.; Tojo, K., Strikingly different electronic spectra of structurally similar perylene imide compounds. *Dyes Pigments* **2006,** *70* (2), 126-135.
(42) Hino, K.; Mizuguchi, J., N,N '-bis 2-(4-pyridyl)ethyl perylene-3,4 : 9,10-bis(dicarboximide) m-creseol disolvate. *Acta Crystallogr. E* **2005,** *61*, O672-O674.
(43) Mizuguchi, J.; Hino, K., N,N '-bis 2-(4-pyridyl)ethyl perylene-3,4 : 9,10-bis(dicarboximide) phenol disolvate. *Acta Crystallogr. E* **2005,** *61*, O669-O671.
(44) Mizuguchi, J.; Tojo, K., Crystal structure of N,N '-bis(3,5-xylyl)perylene-3,4 : 9,10-bis(dicarboximide), C40H26N2O4. *Z. Kristallogr.* **2001,** *216* (3), 375-376.
(45) Mizuguchi, J.; Tojo, K., Electronic structure of perylene pigments as viewed from the crystal structure and excitonic interactions. *J. Phys. Chem. B* **2002,** *106* (4), 767-772.
(46) Hadicke, E.; Graser, F., Structures of 11 perylene-3,4-9,10-bis(dicarboximide) pigments. *Acta Crystallogr. C* **1986,** *42*, 189-195.
(47) Mizuguchi, J.; Shikamori, H., Spectral and crystallographic coincidence in a mixed crystal of two components and a crystal of their hybrid component in pyrrolopyrrole pigments. *J. Phys. Chem. B* **2004,** *108* (7), 2154-2161.
(48) Mizuguchi, J.; Miyazaki, T., Crystal structure of 3,6-bis(4-biphenylyl)pyrrolo[3,4-c]pyrrole-1,4-dione, C30H20N2O2. *Z.Kristallogr.-NCS.* **2002,** *217* (1), 43-44.
(49) Mizuguchi, J., J-aggregate-like structure and its influence on the electronic spectrum of 1,4-diketo-3,6-bis(4-biphenylyl)pyrrolo 3,4-c pyrrole. *J. Imag. Sci. Tech.* **2002,** *46* (3), 257-261.

(50) Mizuguchi, J., A low-temperature phase of the 1 : 1 complex of 2-(6-diethylamino-3-diethyliminio-3H-xanthen-9-yl)benzoate with ethyl gallate at 93 K. *Acta Crystallogr. E* **2008,** *64*, O1238-U1673.

(51) Sato, K.; Shima, H.; Mizuguchi, J., Electronic Spectra of the 1:1 Rhodamine B Base with Ethyl Gallate in Solution and in the Solid State. *J. Imag. Sci. Tech.* **2009,** *53* (5).

(52) Senju, T.; Hoki, T.; Mizuguchi, J., 3,10-Dichloro-5,12-dihydroquino 2,3-b acridine-7,14-dione. *Acta Crystallogr. E* **2006,** *62*, O261-O263.

(53) Senju, T.; Minguchi, J., The electronic structure of C.I. Pigment Red 209. *Dyes Pigments* **2008,** *76* (3), 760-764.

(54) Senju, T.; Nishimura, N.; Hoki, T.; Mizuguchi, J., 2,9-Dichloro-5,12-dihydroquino 2,3-b acridine-7,14-dione (red phase). *Acta Crystallogr. E* **2005,** *61*, O2596-O2598.

(55) Senju, T.; Nishimura, N.; Mizuguchi, J., Polymorph of 2,9-dichloroquinacridone and its electronic properties. *J. Phys. Chem. A* **2007,** *111* (15), 2966-2970.

(56) Senju, T.; Hoki, T.; Mizuguchi, J., The triclinic form of 2,9-dichloro-5,12-dihydroquino 2,3-b acridine-7,14-dithione dimethylacetamide disolvate. *Acta Crystallogr. E* **2005,** *61*, O1930-O1932.

(57) Mizuguchi, J.; Hoki, T.; Senju, T., Polymorph of dimethylacetamide-solvated crystals of 2,9-dichlorodithioketoquinacridone and their hydrogen bonding effect. *Dyes Pigments* **2006,** *69* (1-2), 54-61.

(58) Hoki, T.; Senju, T.; Mizuguchi, J., The monoclinic form of 2,9-dichloro-5,12-dihydroquino 2,3-b acridine-7,14-dithione dimethylacetamide disolvate. *Acta Crystallogr. E* **2005,** *61*, O1927-O1929.

(59) Bernstein, J.; Choshen, E. G., The polymorphic structures of a squarylium dye - the monoclinic (green) and triclinic (violet) forms of 2,4 bis(2-hydroxy 4-diethylaminophenyl)-1,3-cyclobutadienediylium 1,3-diolate. *Mol. Cryst. Liq. Cryst.* **1988,** *164*, 213-229.

(60) Tristanikendra, M.; Eckhardt, C. J., Influence of crystal fields on the quasimetallic reflection spectra of crystals - optical-spectra of polymorphs of a squarylium dye. *J. Chem. Phys.* **1984,** *81* (3), 1160-1173.

(61) Jasperse, J. R.; Kahan, A.; Plendl, J. N.; Mitra, S. S., Temperature dependence of infrared dispersion in ionic crystals LiF and MgO. *Phys. Rev.* **1966,** *146* (2), 526-542.
